# Sound-by-sound thalamic stimulation modulates midbrain auditory excitability and relative binaural sensitivity in frogs

**DOI:** 10.3389/fncir.2014.00085

**Published:** 2014-07-25

**Authors:** Abhilash Ponnath, Hamilton E. Farris

**Affiliations:** ^1^Neuroscience Center, Louisiana State University Health Sciences CenterNew Orleans, LA, USA; ^2^Department of Otolaryngology and Biocommunication, Louisiana State University Health Sciences CenterNew Orleans, LA, USA; ^3^Department of Cell Biology and Anatomy, Louisiana State University Health Sciences CenterNew Orleans, LA, USA

**Keywords:** thalamus, descending modulation, inferior colliculus, binaural, selective attention, torus semicircularis

## Abstract

Descending circuitry can modulate auditory processing, biasing sensitivity to particular stimulus parameters and locations. Using awake *in vivo* single unit recordings, this study tested whether electrical stimulation of the thalamus modulates auditory excitability and relative binaural sensitivity in neurons of the amphibian midbrain. In addition, by using electrical stimuli that were either longer than the acoustic stimuli (i.e., seconds) or presented on a sound-by-sound basis (ms), experiments addressed whether the form of modulation depended on the temporal structure of the electrical stimulus. Following long duration electrical stimulation (3–10 s of 20 Hz square pulses), excitability (spikes/acoustic stimulus) to free-field noise stimuli decreased by 32%, but returned over 600 s. In contrast, sound-by-sound electrical stimulation using a single 2 ms duration electrical pulse 25 ms before each noise stimulus caused faster and varied forms of modulation: modulation lasted <2 s and, in different cells, excitability either decreased, increased or shifted in latency. Within cells, the modulatory effect of sound-by-sound electrical stimulation varied between different acoustic stimuli, including for different male calls, suggesting modulation is specific to certain stimulus attributes. For binaural units, modulation depended on the ear of input, as sound-by-sound electrical stimulation preceding dichotic acoustic stimulation caused asymmetric modulatory effects: sensitivity shifted for sounds at only one ear, or by different relative amounts for both ears. This caused a change in the relative difference in binaural sensitivity. Thus, sound-by-sound electrical stimulation revealed fast and ear-specific (i.e., lateralized) auditory modulation that is potentially suited to shifts in auditory attention during sound segregation in the auditory scene.

## Introduction

Auditory processing is not limited to stimulus driven responses. Indeed, a listener's experience or internal state can modulate auditory sensitivity, implicating a role for descending processing in shaping aspects of the ascending auditory code (Ciocca and Bregman, 1987; Darwin, 2005; Palmer et al., 2007; Shinn-Cunningham, 2008; Bajo et al., 2010; Fritz et al., 2010; Shamma et al., 2011; Grimsley et al., 2013). For example, in psychoacoustic and gross electrophysiological studies, subjects exerting selective attention can bias or limit neural coding to specific acoustic sources among many (Alain and Arnott, 2000; Carlyon et al., 2001; Elhilali et al., 2009; Kerlin et al., 2010; Besle et al., 2011; Varghese et al., 2012; Hairston et al., 2013). The result is modulated processing of subsets of ongoing stimuli in a flexible or “on-demand” manner, such that groups of neurons may exhibit quick (<100 ms) shifts in sensitivity from one stimulus (or subset) to another, including for complex sounds (Koch et al., 2011; Mesgarani and Chang, 2012; Lakatos et al., 2013).

With respect to assays at the single cell level, several studies have shown descending modulation of auditory sensitivity of single units in the midbrain, thalamus and cortex following electrical stimulation of cortical and thalamic nuclei (Suga et al., 1997, 2002, 2003; Zhang and Suga, 1997, 2000; Zhang et al., 1997; Ma and Suga, 2001; Xiao and Suga, 2002; Wu and Yan, 2007). Although such modulation may be quite specific in shifting sensitivity to certain stimulus attributes (e.g., frequency resolution), the temporal parameters of the modulatory stimuli and their subsequent effects are often much longer than the fast acoustic changes typically attended to in the auditory scene. Indeed, modulatory electrical stimuli used in many experiments are often designed to elicit long-term plasticity that is similar to auditory learning (Weinberger, 1998; Gao and Suga, 2000). Thus, descending stimulation on the order of seconds to minutes causes modulation of sensitivity lasting similar durations or longer (Yan and Ehret, 2002; Wu and Yan, 2007). This time frame contrasts the “on-demand” modulation suggested during behavioral assays (Koch et al., 2011; Hairston et al., 2013). Building on these studies of long-term modulation, experiments here measured the modulatory effects of both long-term (seconds) and short-term (ms; sound-by-sound) thalamic stimulation on the auditory responses of single cells. In particular, we assessed whether sensitivity can be modulated on a time scale closer to that found for changes in the auditory scene and whether a large difference in the temporal parameters of modulatory stimuli can affect both the duration of modulation and its form (e.g., increased or decreased excitability).

Although descending modulation of ascending processing can occur within and between many processing nodes (e.g., nuclei) in the vertebrate auditory system (Yan and Ehret, 2002; Suga, 2008; Castellano-Munoz et al., 2010; Rabbitt and Brownell, 2011; Bajo and King, 2012; Hairston et al., 2013), our study focused on the auditory midbrain of frogs; in particular the torus semicircularis (TS). Like its homolog, the mammalian inferior colliculus (Bass et al., 2005; Wilczynski and Endepols, 2007), the TS receives both ascending (Luksch and Walkowiak, 1998), and descending input (Feng and Lin, 1991; Gonzalez and Smeets, 1991; Endepols and Walkowiak, 2000; Endepols et al., 2000; Casseday et al., 2002). Whereas the ascending circuitry at this level of the midbrain is relatively well studied across taxa (Klug et al., 1995; Alder and Rose, 1998, 2000; McAlpine et al., 1998; Leroy and Wenstrup, 2000; Casseday et al., 2002; Edwards and Rose, 2003; Portfors and Sinex, 2005), research on the function of descending circuitry is largely limited to work with mammals (Yan and Suga, 1996a,b, 1998, 1999; Zhang and Suga, 1997, 2000; Zhang et al., 1997; Hurley and Pollak, 1999, 2001, 2005; Suga et al., 2000; Hurley et al., 2002; Hurley, 2007; Wu and Yan, 2007; Rinne et al., 2008; Melo et al., 2009). With respect to frogs, while the chemoarchitecture of the descending input appears similar to that in mammals (Feng and Lin, 1991; Marin et al., 1997; O'Connell et al., 2010), there are few data showing descending modulation, with only *in vitro* (brain removed) electrical stimulation of the striatum and thalamus shown to alter responses of TS neurons to ascending electrical stimulation of the auditory nerve (Endepols and Walkowiak, 1999, 2000). Thus, it is still unknown how endogenous descending circuitry affects processing of acoustic stimuli, especially those stimuli important to communication. Understanding this is critical because the TS is hypothesized to be an interface between the auditory and motor systems (Schmidt, 1988, 1990; Strake et al., 1994; Endepols et al., 2003), potentially responsible for selecting the stimuli to be matched with the appropriate social behavior (Hoke et al., 2008).

## Materials and methods

The general preparation and recording procedures follow those used by Ponnath et al. (2013).

### Animals

Frogs, *Rana pipiens* (*N* = 86; yielding 264 analyzed cells) and *Hyla cinerea* (*N* = 8; yielding 18 analyzed cells), were supplied by licensed commercial vendors. Whereas cells recorded in *R. pipiens* were used in all experiments, cells recorded in *H. cinerea* were only included in two experiments testing modulation of the auditory response to noise (see below). Prior to euthanasia, each individual was used in only one set of experiments (e.g., long-term, sound-by-sound or ear specific modulation). All animal care and experimental procedures were performed in accordance with the protocols established by the veterinarians on the Louisiana State University Health Sciences Center Animal Care and Use Committee.

### Preparation and recording

Following general anesthesia (2.5% urethane immersion, 10–20 min), the midbrain and thalamus of adult frogs were exposed by resecting a small piece of skull dorsal to the optic tectum. Topical analgesia of the surgical area (from the start of surgery to the end of testing) was accomplished using dibucane cream (0.9%). The head wiping reflex was monitored as an indicator of anesthesia effectiveness. After 24 h of recovery from anesthesia, frogs were immobilized by intramuscular injection of succinylcholine chloride (22 μg/g body weight; Glagow and Ewert, 1997). A moist towel maintained cutaneous respiration, while a digital thermometer monitored temperature. Immobilized frogs were mounted dorsal side up on an air table in a foam (Tecnifoam 4 in.; NRC 1.21 at 500 Hz) lined Faraday cage that was closed on all sides. The frog's mouth was propped open >0.25 cm, so that differences in directional sensitivity were limited to binaural neural mechanisms (Feng and Shofner, 1981; Pinder and Palmer, 1983). Extracellular electrophysiological activity was recorded using thin-walled glass micropipettes filled with 4 M NaCl (3–10 MΩ). After amplification (GRASS P511 with high impedance head stage) neural responses were digitized (100 μs sampling period) using a TDT AD3 and System II array processor with custom written software. Auditory units were isolated (peak action potential voltage was >20 dB above the noise at recording start) using a series of search stimuli that cover the frequency range of auditory sensitivity and call spectra in both species (~0.1–4 kHz) (Liff, 1969; Mecham, 1971; Mudry et al., 1977; Fuzessery and Feng, 1982, 1983; Moss and Simmons, 1986; Ronken, 1991; Goense and Feng, 2012). These stimuli included (but were not limited to) a 60-ms Gaussian noise, 30-ms tones; a 20-ms band limited noise centered at 2 kHz; and segments of *R. pipiens* calls (see below). As done previously (Ponnath et al., 2013), the recording site was recovered using the stereotactic position (i.e., electrode depth) measured with a calibrated micromanipulator (Narashige) and electrolytic lesions. Electrodes were inserted in the medial half of the tectum with recording sites ranging across the dorsal-ventral depths of the principle nucleus of the TS. Animals were euthanized using the external anesthesia agent (urethane immersion).

### Acoustic stimulus production

Acoustic stimuli were generated and amplified using a TDT II DA3 16 bit D-A converter (40 μs sample period) and a Harmon/Kardon integrated amplifier, respectively. Stimulus amplitude was controlled with TDT PA2 programmable attenuators. For free field experiments, acoustic stimuli were presented from a single Fostek #FE127 broadband speaker positioned at 0° normal to the front of the frog (30 cm distance). For dichotic acoustic stimulation of binaural units, sounds were presented at 1 mm from each tympanum using Etyomotic (ER 4 Micropro) headphones attached to 1 cm length plastic tubes. Note that the headphones were not present during experiments with freefield stimulation. All speakers were calibrated using a Bruel and Kjaer B&K 2608 measuring amplifier with a B&K model 4133 1/2-in. microphone and a B&K 4220 pistonphone calibrator. Free field calibration was done at the position of the recording site, directly between and dorsal to the two tympana. Each headphone was independently calibrated 1 mm from the tube. Because the tubes were not sealed to the head, headphones were also calibrated at the contralateral ear, showing that external propagation resulted in >21 dB attenuation at the contralateral tympanum. Symmetry between the two headphones was ±1.5 dB at all frequencies. All frequency components of the ambient noise were measured to be ≤21.5 dB SPL (peak ambient noise range 120–230 Hz). The peak amplitude of all stimuli was 90 dB SPL.

### Electrical stimulation

**Figure 1:**
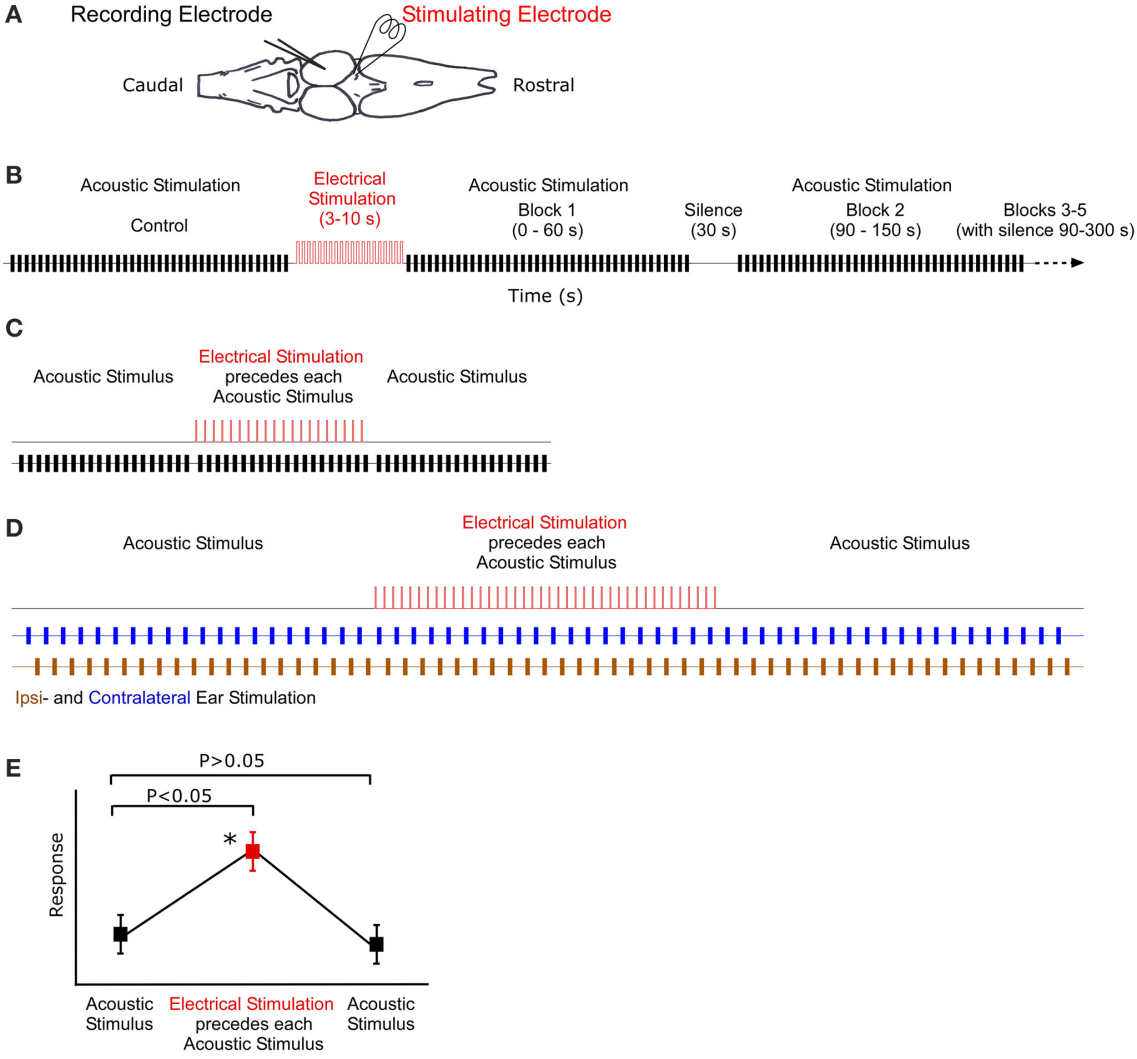
**Illustration of recording, stimulation and analysis techniques. (A)** Dorsal view outline of the frog brain. Recording and stimulating electrodes are in the midbrain and thalamus, respectively. **(B)** Stimulus protocol for long-term stimulation. Control block of acoustic stimuli (black) are followed by 10 s of electrical stimulation (red). Subsequent blocks of acoustic stimuli (and silence) follow at particular intervals. **(C)** Sound-by-sound electrical stimulation protocol. Tests consisted of three blocks of acoustic stimuli. A single electrical pulse (2 ms duration) preceeded (25 ms) each acoustic stimulus in the second block. **(D)** Antiphonal dichotic stimulation using headphones to stimulate both ears, ipsi- and contralateral to the recording site. Monotic pulse period is 2 s. After 20 pulses to each ear, the thalamus is electrically stimulated prior to each acoustic stimulus. Subsequently, the acoustic stimuli continue, creating three analysis blocks: before, during and after electrical stimulation. **(E)** Hypothetical data showing the two analytic criteria for a significant modulatory effect of sound-by-sound electrical stimulation. For modulation, responses during electrical stimulation must be different from control responses before, whereas responses after electrical stimulation must not (Tukey test for multiple comparisons). Asterisk marks significant modulation.

*In vivo* electrical stimulation is modeled after that used to modulate auditory circuitry *in vitro* (Endepols and Walkowiak, 2000). In the thalamic lobe ipsilateral to the recording electrode, a bipolar stimulating electrode was inserted below (500–800 μm, *R. pipiens*; 300–400 μm, *H. cinerea*) the dorsal surface of the caudal portion of the thalamus (i.e., overlapping the rostral most portions of the tectum) (Figure 1) and lateral to the third ventricle. This position targets the posterior and central thalamic nuclei; both contain descending projections to the laminar and principal nuclei of the TS (Feng and Lin, 1991; Wilczynski and Endepols, 2007). The stimulating electrode (FHC Inc.) was either concentric (50 μm pencil tip) or parallel (200 μm spacing). There was no difference in recordings when using these two models. Electrical stimuli were produced by a Grass SD9 stimulator gated by a TTL pulse.

### Long-term thalamic stimulation

Long-term electrical stimulation is operationally defined as electrical input that is temporally uncorrelated to acoustic stimuli and an order of magnitude longer in duration than functional call stimuli (Larson, 2004; Velez and Bee, 2013). To test whether such stimulation can modulate auditory sensitivity, the following sequence of stimuli was used (Figure 1B). (1) 20 repetitions of silence (400 ms duration) to quantify any spontaneous activity. (2) 40 repetitions of Gaussian noise (200 ms duration 0.1 ms cosine ramps) established the unmodulated acoustic response of the unit. The noise buffers changed for each repetition. (3) Thalamic electrical stimulation for 3–10 s (a train of 2 ms duration DC pulses of 6 V at 20 Hz repetition rate). (4) Following electrical stimualtion, 200 ms duration pulses of Gaussian noise were presented in blocks of 40. Each block lasted 60 s and started at 0, 90, 240, 510, and 870 s post electrical stimulation. Unless noted otherwise, recording buffers were 400 ms long, with a 1.5 s stimulus period. Any spontaneous activity during the interblock silences (durations: 30, 90, 210, and 300 s, respectively) was also recorded in repeated 400 ms sweeps every 1.5 s; spontaneous activity was quantified in the manner that acoustically driven responses were (spikes/400 ms sweep). During acoustically driven responses, spontaneous activity correction was accomplished by subtracting the mean spontaneous rate recorded in the silent interval preceding each response block. This allowed for an updated spontaneous rate correction. Note that in order for a cell to later be analyzed for modulation of spontaneous activity, it had to exhibit action potentials in at least 4/20 silent sweeps, which is statistically different from 0. Prior to statistical analysis, auditory response rates for the different time points post-electrical stimulation were normalized within cells to the response rate measured prior to electrical stimulation. This provided a relative change in response rate. Responses were analyzed using a two tailed t-test (adjusted for multiple comparisons) comparing auditory sensitivity pre- and post-electrical stimulation.

For analysis of the temporal effects of modulation, spike trains were delinieated into three different temporal response windows. The initial window (IW) and the sustained window (SW) consisted of times 0–30 and 30–400 ms of the spike train, respectively. The total window (TW) was the entire 400 ms recorded buffer.

### Sound-by-sound thalamic stimulation

Using *R. pipiens* only and different individuals from those in the long-term modulation tests above, short-term modulation was measured using free field acoustic stimuli paired with electrical pulses presented in the following protocol (Figure 1C). (1) 20 repetitions of silence (400 ms duration recording; 1.5 s stimulus period) quantified any spontaneous activity. (2) 20 repetitions of Gaussian noise pulses (200 ms duration; 1.5 s period) established the unmodulated acoustic response of each auditory unit. (3) 20 repetitions of paired electric and acoustic stimulation consisting of a single electrical pulse (2 ms, 6 V) 25 ms before a single 200 ms Gaussian noise pulse. (4) 20 repetitions of Gaussian noise pulses (200 ms duration; 1.5 s period) to assess whether responses returned to the pre-modulated rate. (5) 20 repetitions of a single electrical pulse paired with acoustic silence (1.5 s period) to determine whether any action potentials were electrically (i.e., not acoustically) driven. Note, however, that the 25 ms interval between electrical and acoustic stimulation was an order of magnitude longer than the latency for electrically driven spikes (data not shown), allowing for clear delineation between electric and acoustic responses during the combination stimuli, as well. Any unit that exhibited electrically driven responses was not included in the analysis.

Following the above protocol the Gaussian noise pulses were replaced with one of three *R. pipiens* male calls (Mecham, 1971; Larson, 2004) to assess whether sound-by-sound electrical stimulation can modulate sensitivity to functional stimuli (excerpts from the Library of Natural Sounds, Cornell University): two forms of the so-called “Grunt-Chuckle” and a “Snore.” Based on Larson (2004), the recording of the Snore was filtered (bandpass 220 Hz–2.7 kHz) to remove background noise and subsequently multiplied by the original envelope (Hilbert transform) to maintain its temporal characteristics. The durations of these complete calls (1.97, 1.37, and 0.75 s, respectively; 44100 Hz sample rate) necessitated a change in the duration of the recordings. Responses were recorded over 2.1 and 1.5 s for the Grunt-Chuckles (stimulus period 2.5 s) and 1.1 s for the Snore (stimulus period 1.5 s). As with noise above, the time between the sound-by-sound electrical pulse and the call stimuli was 25 ms, except for the snore, in which it was 200 ms.

For all sound-by-sound stimulus experiments, modulation was deemed to occur (within a cell) if there was (1) a significant difference between responses before and during electrical stimulation; and (2) a non-significant difference between responses before and after electrical stimulation. This latter test confirms that responses return to the premodulated level (Figure 1E). Statistical significance was tested with a Tukey's test for multiple comparisons (SAS Institute Inc.).

### Ear specific sound-by-sound modulation

In contrast to free field experiments, earphones (Ponnath et al., 2013) were used to test whether thalamic electrical stimulation could modulate binaural auditory sensitivity in an ear specific manner. After isolating a binaural unit using dichotic search stimuli, the following stimulus sequence was presented (Figure 1D). (1) Alternating dichotic sequence of 20 Gaussian noise pulses (200 ms duration) to each ear (40 pulses total). Pulse period was 1 s (or 2 s within each ear). (2) Alternating dichotic sequence of 20 Gaussian noise pulses as in stimulus set 1. Each pulse was preceded (25 ms) by a single DC electrical pulse (2 ms, 6 V) to the thalamus. (3) Stimulus set 1 was repeated. Each noise pulse differed. Relative binaural sensitivity, a measure of stimulus discriminability (*d*′), is calculated as the difference between the mean response to sounds at each ear divided by the mean response standard deviation.

## Results

For single speaker, free field acoustic stimulation coupled with long-term or sound-by-sound electrical stimulation, statistically significant modulation of auditory responses was measured in 37.2% (*N* = 89 of 239) of cells recorded in the TS. When tested with a single 200 ms Gaussian noise pulse, the spike train responses of modulated cells were not limited to particular post stimulus time histogram (PSTH) types and included cells with phasic (28.7%), primary-like (33.3%), pauser (14.9%), or tonic (23.0%; including chopper-like) responses (Pfeiffer, 1966; Shofner and Young, 1985).

### Long-term thalamic stimulation

**Figure 2:**
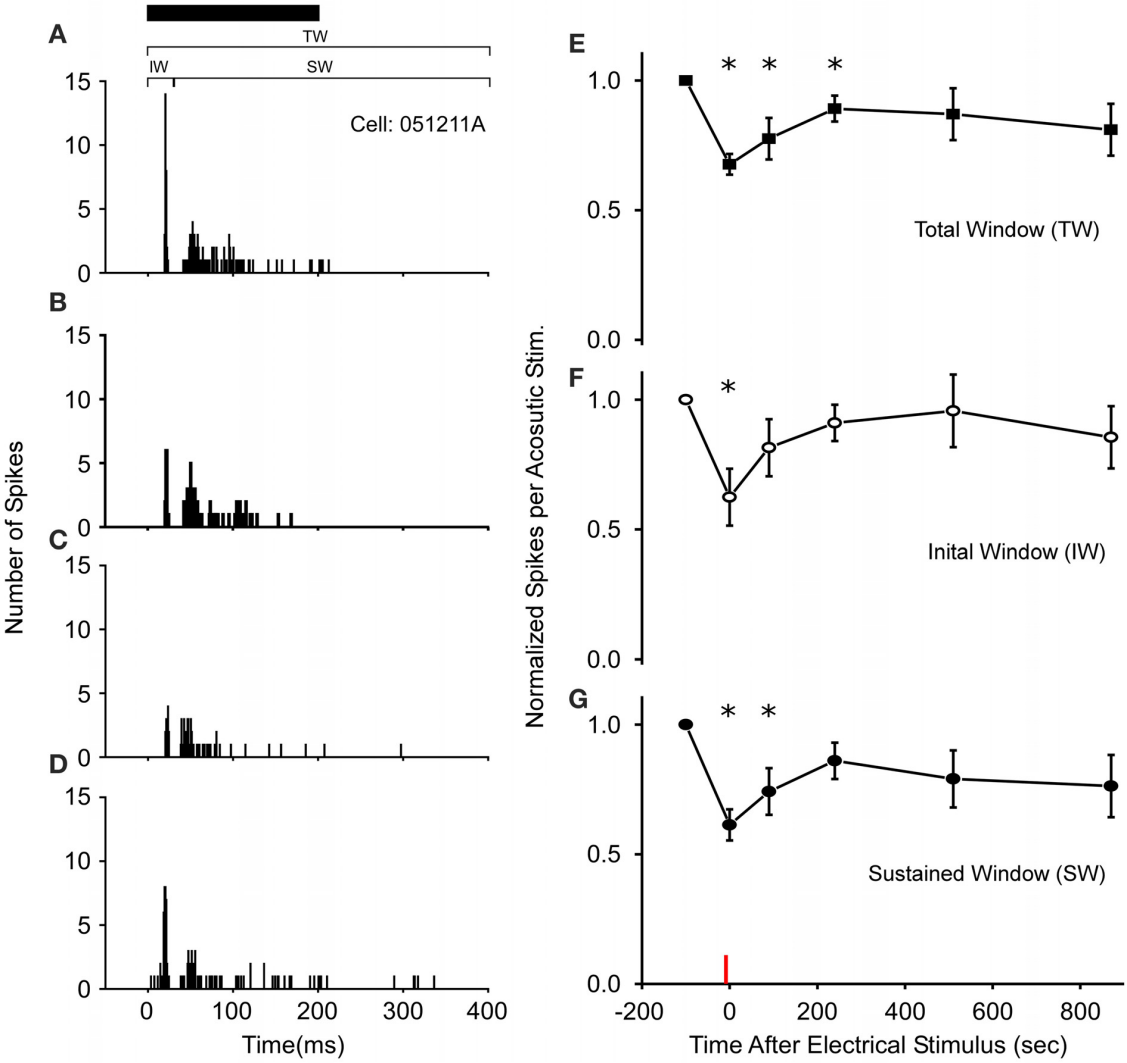
**Long-term thalamic stimulation decreases acoustic sensitivity for several minutes**. **(A)** PSTHs of a cell's response to a 200 ms noise (black bar). Following 10 s of electrical stimulation (2 ms DC pulses, 20 Hz, 6 V) the number of spikes/acoustic stimulus in both the initial (IW) and sustained windows (SW) was reduced immediately **(B)** and after 90 s **(C)**. **(D)** 510 s post electrical stimulation the response increases toward control levels. PSTHs are for 40 presentations. **(E–G)** Population wide response (*N* = 18 cells) for each time window of the spike train when normalized to control levels. Asterisks note statistically significant response reductions. Red line denotes time of electrical stimulation. PSTH bandwidth is 1 ms.

Following 3–10 s of thalamic stimulation, all cells (*N* = 18 of 57 tested) exhibiting auditory modulation showed reduced excitability to the Gaussian noise pulses for several minutes (Figures 2A–D). In particular, the average number of spikes/acoustic stimulus across the total spike train (Figure 2E) was reduced by 32 ± 4% immediately following electrical stimulation and remained below the control levels for up to 3 min. Subsequently, responses returned to near pre-modulated rates, as the mean and variance in auditory responses slowly increased. This reduction in total response in the first 3 min post-electrical stimulation was not due to modulation of a particular segment of the response, as both the initial and SWs of the spike train exhibited significantly reduced auditory excitability (Figures 2F,G). Recordings in *R. pipiens* and *H. cinerea* contributed 16 and 2 modulated cells to the dataset, respectively. The small sample of cells from *Hyla* were quite similar to those in *Rana* in their reduction in sensitivity at each time point and were thus, included (*P* > 0.7 at each time point; also, each *Hyla* cell's modulated response was within one standard of deviation of the mean *Rana* response).

### Sound-by-sound thalamic stimulation

**Figure 3:**
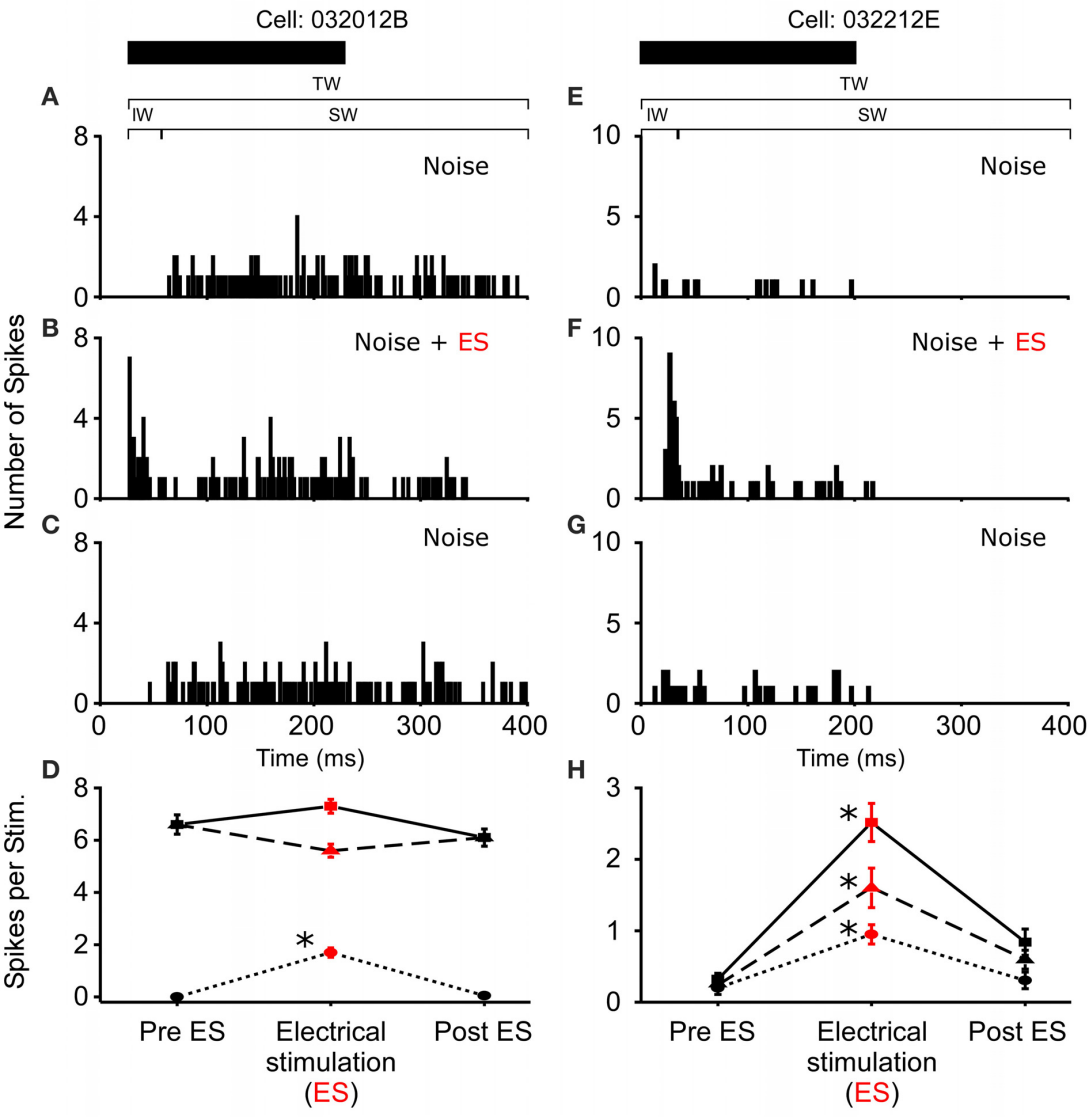
**Increased excitability due to sound-by-sound electrical stimulation**. **(A–C)** Example cell showing increased auditory excitability in the initial window (IW) when acoustic stimuli are coupled with electrical stimulation of the thalamus. The spike train changes from a tonic responses to a primary like response. **(E–G)** Example cell in which electrical stimulation increases the auditory response in all segments of the spike train. PSTHs are for 20 presentations. **(D,H)** For each column of PSTHs, symbols and lines represent the different temporal windows: IW (circle; dot); SW (triangle; dash); TW (square; solid). Asterisks note statistically significant modulation.

**Figure 4:**
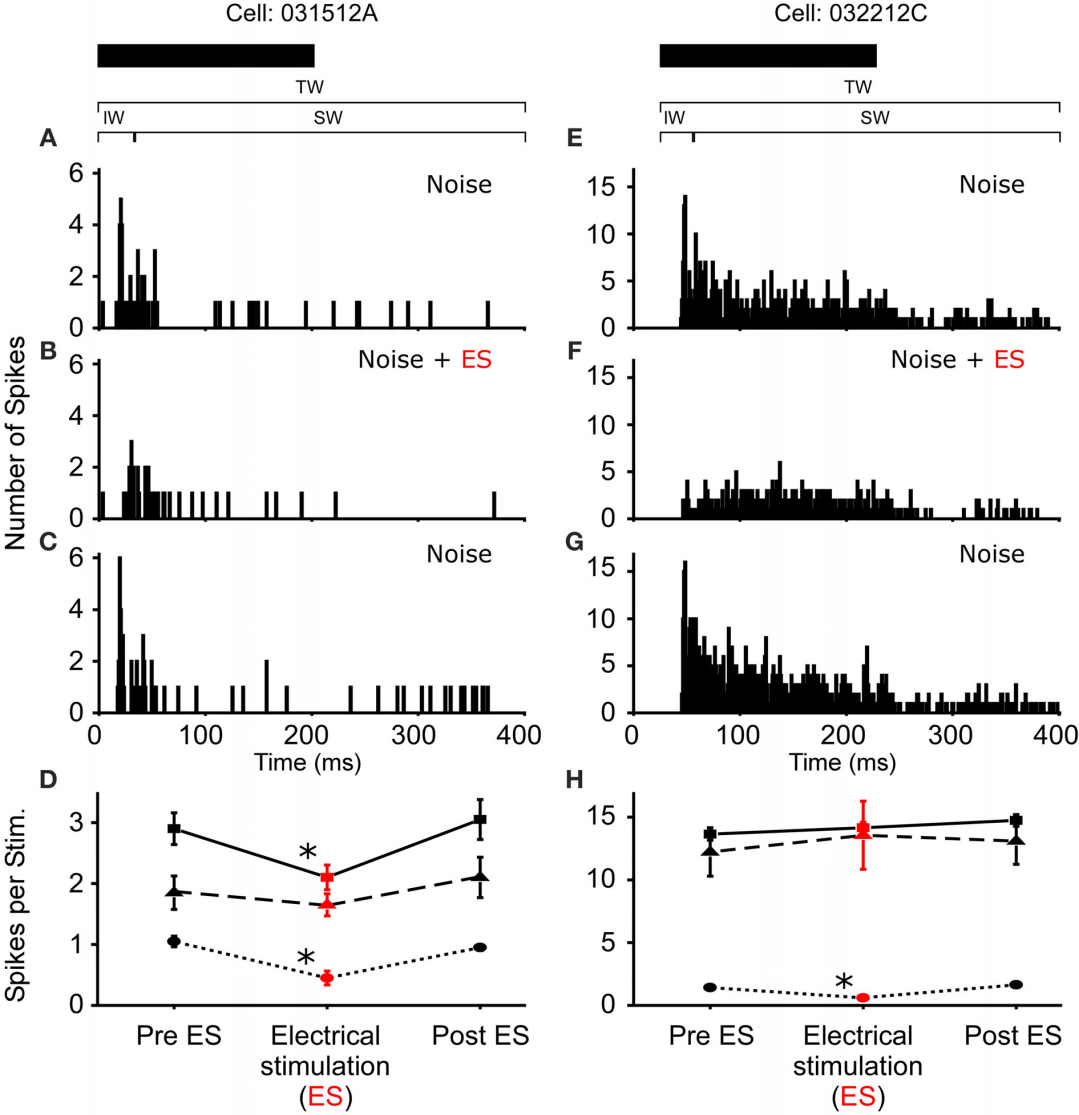
**Reduced excitability due to sound-by-sound electrical stimulation**. **(A–C)** PSTHs for a cell exhibiting a reduced response in multiple segments of the spike train. **(E–G)** Reduced response is found in the initial window, with the other windows showing no significant change. Modulation switches the cell's response from primary-like to tonic. PSTHs are for 20 presentations. **(D,H)** For each column of PSTHs, symbols and lines represent the different temporal windows: IW (circle; dot); SW (triangle; dash); TW (square; solid). Asterisks note statistically significant modulation.

**Figure 5:**
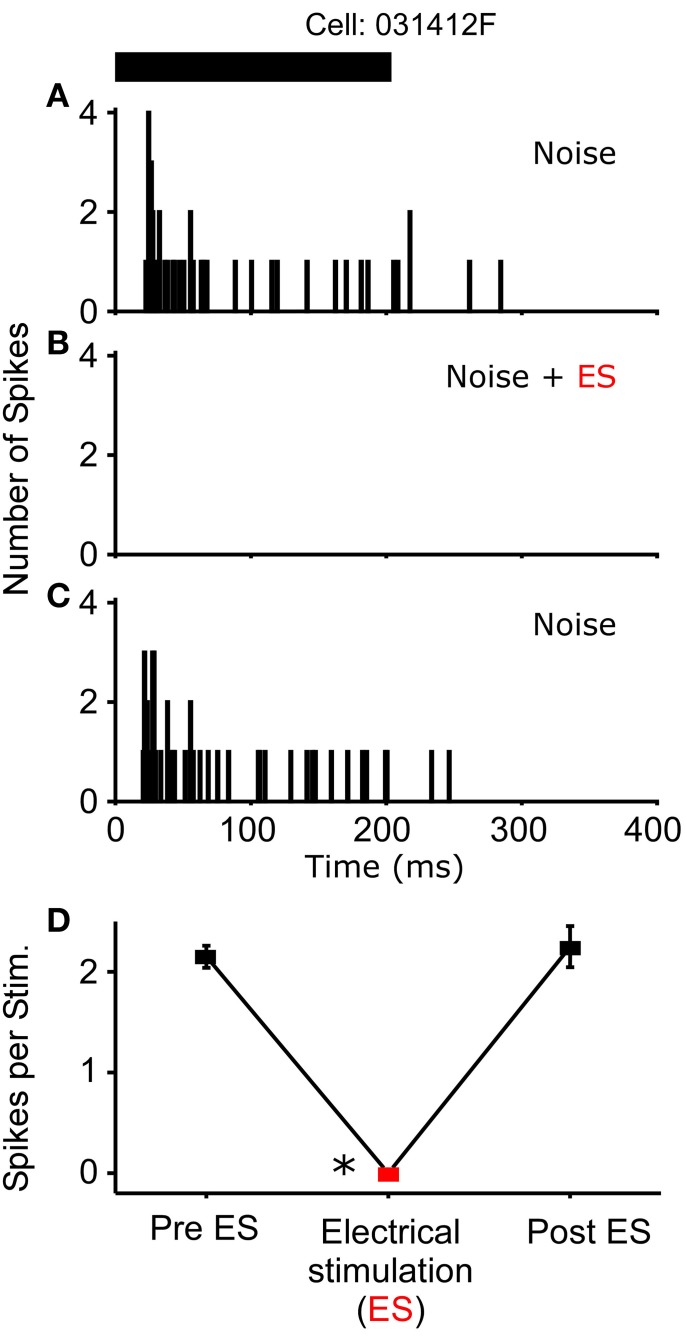
**One cell exhibited a complete loss of its auditory response during sound-by-sound electrical stimulation**. Panels are as in Figures 3, 4. Asterisk notes statistically significant modulation.

In *R. pipiens*, off the 171 cells tested using sound-by-sound electrical stimulation and free field acoustic noise stimuli, 51 exhibited modulation. In contrast to the consistent reduction in auditory excitability following long-term thalamic stimulation, this form of electrical stimulation resulted in two different modulatory effects on the number of spikes/stimulus: increased (*N* = 38; Figure 3) and decreased excitability (*N* = 13; Figures 4, 5). For example, Figure 3 shows the PSTHs for two auditory units presented with the experimental stimulus series: acoustic, acoustic + electrical, and acoustic stimuli, respectively. Here the addition of sound-by-sound electrical stimulation increased auditory excitability, with such increases resulting from either a limited change in a single time window (Figure 3D) or from a more broad increase across all segments of the spike train (Figure 3H). The mean (±STD) relative increase in excitability for the total window (response during electrical stim./response pre-electrical stim.) was 1.86 ± 1.22 (range: 1.06–7.82). It is important to note that the excitation (i.e., action potentials) measured in these cells was in response to the acoustic stimuli and not electrical stimulation alone, as the failure of electrical pulses by themselves to elicit action potentials enabled the exclusion of non-acoustic responses from the dataset. In contrast to excitation, Figures 4, 5 show examples of cells in which there was a reduction in sensitivity. Whereas Figures 4A–D show a modulatory effect that is significant for the initial and total spike train windows, Figures 4E–H show the response of a cell that was modulated only in the onset portion (i.e., IW) of the spike train, converting the response from primary-like to tonic. Although the vast majority of reduced responses were partial (i.e., limited to a portion of the response to the acoustic stimulus; 12/13 cells), the one case in which reduced excitability was 100% is shown in Figure 5. Overall, the mean relative change (±STD) in spikes/stimulus in cells showing decreased auditory excitability was 0.55 ± 0.24 (range: 0.83–0). *Post-hoc* ANOVA analysis in which stimulus repetition number (i.e., 1–20) was nested within cell and stimulus block (acoustic or acoustic + electric) confirmed that these reductions in response were not due to either a buildup of adaptation or modulation (effect of repetition number on spikes/stim. *P* > 0.25). The analysis found only an effect due to the presence of electrical stimulation (*P* < 0.001). This suggests that the interstimulus interval of 2 s was long enough to ensure independence of each presentation of the stimuli, such that the probability of response and modulation on any presentation did not differ. The analysis also provides independent confirmation of the method used in determining the difference between modulated and control conditions (Figure 1E).

**Figure 6:**
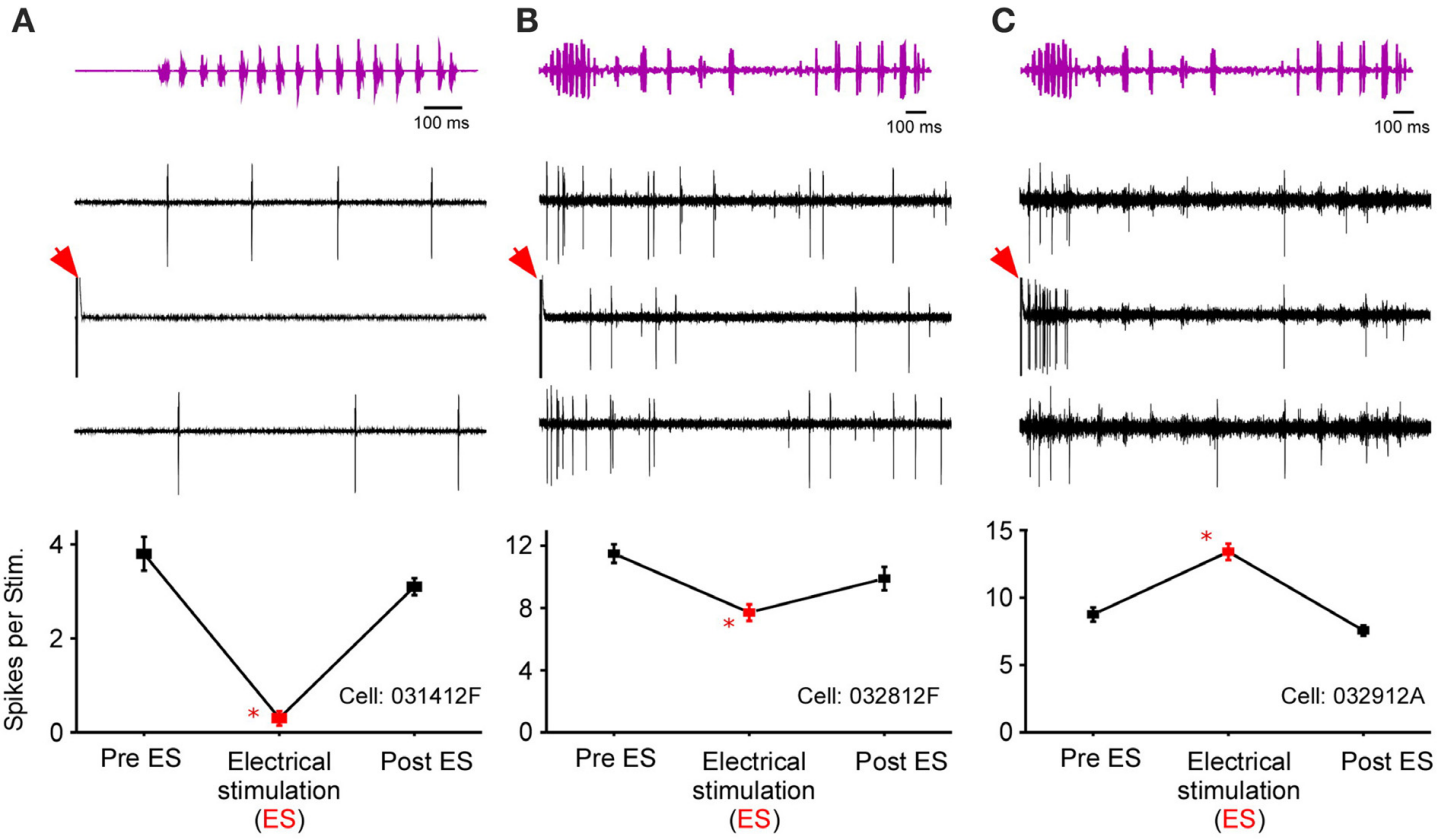
**Responses of three cells to call stimuli before, during, and after thalamic stimulation**. Top row shows the time waveform for the calls [**(A)**, snore; **(B**,**C)**, Grunt-Chuckle]. Middle row shows voltage traces for the three exemplar auditory units. The bottom row summarizes the modulation in the number of spikes per stimulus. Red arrows mark electrical stimulation artifact. Asterisk notes statistically significant modulation.

After testing for sound-by-sound modulation of responses to acoustic noise, 131 cells in *R. pipiens* were held long enough to also test for modulation of responses to one or more functional stimuli (i.e., male calls). Like the modulation of responses to noise, modulation of responses to calls included increases and decreases in excitability (Figure 6). Due to the varied and overlapping acoustic structures of these call stimuli, the experiments here were not able to determine which acoustic parameter is most likely modulated for each cell. However, comparisons across responses show that the likelihood of modulation of the response to one stimulus did not predict modulation of the response to another stimulus: modulation of the response to the 200 ms Gaussian noise, for example, is at best a 21% predictor of modulation to any of the calls (Table 1).

**Table 1:** **Within cell probability that electrical stimulation of the thalamus modulated the responses to two different stimuli**.

**Acoustic stimuli**	**Noise**	**Snore**	**Grunt-Chuckle 1**	**Grunt-Chuckle 2**
Noise	–	0.21 (38)	0.11 (37)	0.21 (33)
Snore	0.47 (17)	–	0.33 (12)	0.09 (11)
Grunt-Chuckle 1	0.31 (13)	0.40 (10)	–	0.36 (11)
Grunt-Chuckle 2	0.54 (13)	0.07 (14)	0.29 (14)	–

*Given that there is significant modulation (either increase or decrease) of the response to stimuli in the left column, numbers show the probability that there is also modulation of responses to stimuli in the four columns to the right. The table is not symmetrical because the likelihood of modulation to each stimulus differed. Numbers in parentheses are the number of cells tested with each stimulus pair. The specific stimulus attributes being modulated were not tested in this experimental design*.

**Figure 7:**
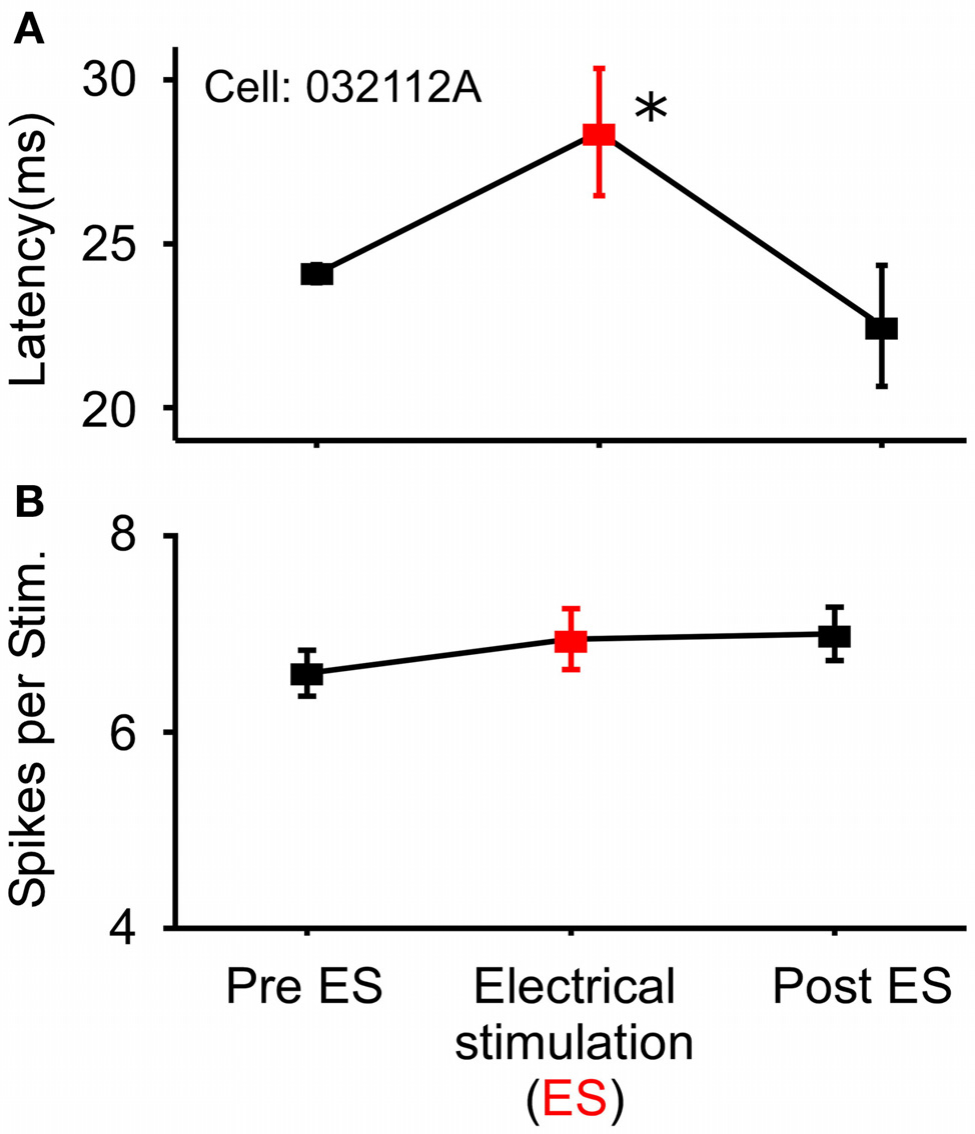
**Example cell exhibiting modulation of response latency**. **(A)** Significant increase in first spike latency during sound-by-sound thalamic stimulation (red symbols) without change in the number of spikes/stimulus **(B)**. Asterisks note statistically significant modulation.

In a small number of cells (*N* = 18) that showed no modulation in the number of spikes/stimulus to noise stimuli, sound-by-sound electrical stimulation modulated the first spike latency by delaying its onset. Using the same statistical criteria for a significant change in spike number (Figure 1), Figure 7 shows a first spike latency shift (and return to control levels), but not a change in spike number. The mean relative latency delay across these cells was 27.3% (±27.6). Note that because spike number did not change, the change in latency was not due to a loss of the first spike, but rather to a delay of the entire spike train.

### Ear specific sound-by-sound modulation

**Figure 8:**
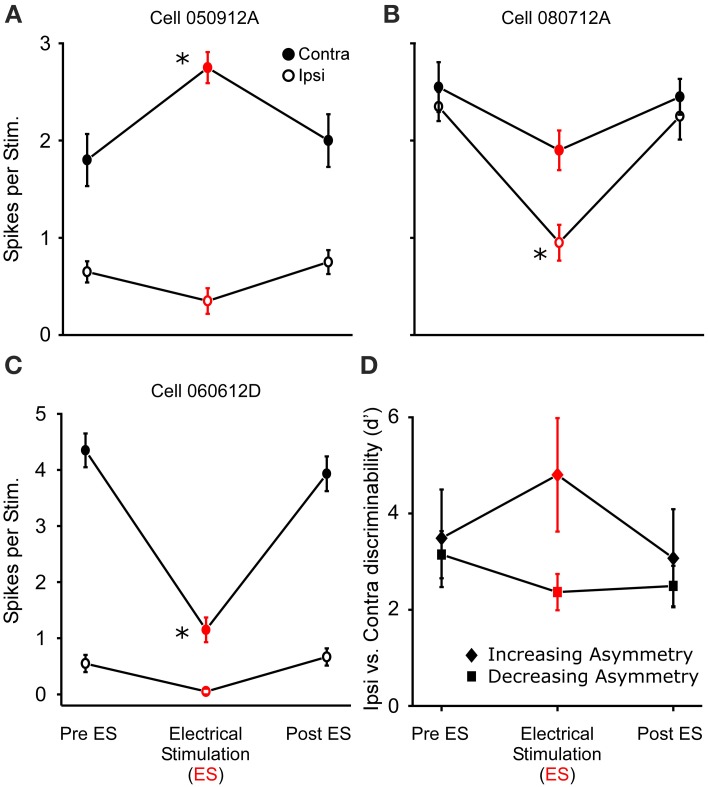
**Thalamic stimulation causes different relative modulation of binaural sensitivity to ipsilateral and contralateral (re. electrical stimulation site) sounds. (A–C)** Sensitivity to ipsi and contralateral acoustic stimulation before during and after electrical stimulation in three separate cells. Each cell shows greater excitation to the contralateral sound. **(A,B)** Electrical stimulation causes an increase in relative binaural sensitivity. **(C)** Decrease in relative binaural sensitivity. **(D)** Summary of 11 and 7 cells showing increased (diamond) and decreased (square) relative sensitivity, respectively (plotted as *d*′). Asterisks note statistically significant modulation. Examples of ear specific modulation in voltage and histogram records are shown in Supplementary Materials 1 and 2, respectively.

In 18 of the 33 binaurally sensitive cells tested in *R. pipiens* and *H. cinerea* combined, presentation of dichotic noise bursts with sound-by-sound thalamic electrical stimulation revealed asymmetric modulatory effects on the responses to sounds at each ear (Figure 8), such that the relative sizes and direction of modulation differed (Figures 8A–C). Thus, to analyze modulation of binaural sensitivity, responses were compared between ears (within cells) to assess a change in relative binaural sensitivity. Eleven neurons showed an increase in relative binaural sensitivity (Figures 8A,B), meaning binaural auditory responses during modulation showed greater sensitivity bias to one ear. The remaining seven neurons showed a decreased sensitivity bias to one ear (Figure 8C). Figure 8D shows the mean relative change in binaural discriminability (*d*′) after segregating cells into these two response categories. The type of change in *d*′ (i.e., increase or decrease) was not dependent on the pre-modulated relative binaural sensitivity (Pre ES *d*′), as the ipsi vs. contra sensitivity was not correlated to the two different types of binaural modulation (Pre ES *d*′ = 3.6 ± 1.1 and 3.0 ± 0.6 for increasing and decreasing binaural modulated cells, respectively; Figure 8D). Cells were included in this dataset if there was significant modulation of the responses to sounds at one ear (*N* = 16; 2 showed significant modulation of responses to both ears). There was no significant difference (*P* = 0.16) in the mean size of modulation (i.e., change in binaural *d*′ following electrical stimulation) between cells modulated in *Rana* (*N* = 12; *d*′ = 4.3 ± 14.9) and those in *Hyla* (*N* = 6; *d*′ = 2.4 ± 2.6).

### Effect of on spontaneous rate

Although a correction was applied to remove spontaneous activity from the responses to acoustic stimuli (see Methods), electrical stimulation during silence provided information on whether the effect of modulation was specific to stimulus processing or the general excitability of the cell (i.e., the set point of the system). For long-term modulation, 19 cells exhibited spontaneous activity in silent control periods before and after the electrical stimulation (eight of these also exhibited auditory modulation and are part of the dataset in Figure 2). When controlling for individual cells (i.e., within cell comparison), and the time after electrical stimulation, GLM analysis showed that the effect of long-term electrical stimulation on spontaneous activity differed significantly from the effect on acoustically driven responses (*F* = 19.2; *P* < 0.0001). This difference is largely driven by the 8 cells which show auditory modulation but no modulation of spontaneous activity (*F* = 14.68; *P* = 0.0005 for the these eight cells only). For sound-by-sound modulation there was no modulation of spontaneous activity by electrical stimulation (*N* = 59; *P* = 0.5842).

## Discussion

In this study, the methods for inducing auditory modulation were relatively gross in that no particular cell types in the thalamus were identified during electrical stimulation. Additionally, the dimensions of the electrical field around the stimulating electrode could not be controlled, potentially allowing for the stimulation of multiple descending circuits related to auditory processing (Wilczynski and Endepols, 2007; Bajo and King, 2012). Nevertheless, the technique reliably elicited modulation of sensitivity to functionally important signals in an unanesthetized preparation. Thus, the experiments were able to reveal two characteristics of modulation that are important to understanding how descending input may contribute to source (and sound) specific processing in frogs, in particular, and vertebrates, in general.

The first characteristic is the potential relationship between the temporal pattern of electrical stimulation and the duration and type of modulation induced: longer, repetitive electrical stimuli caused long duration reductions in sensitivity; and sound-by-sound electrical stimuli caused varied, short-term changes in sensitivity. The results raise the possibility that the mechanisms of plasticity can differ with the amount and/or duration of descending activity, a phenomenon similar to that for aspects of synaptic plasticity (LTP/LTD) (Caporale and Dan, 2008). Thus, to build a framework for understanding how modulation can affect auditory processing, the pattern of the descending code must be included with data describing the anatomy, pharmacology and chemoarchitecture of the descending circuit. From a functional point of view, the range of modulation produced by these two temporal patterns of stimulation suggest a variety of different roles for descending input during auditory processing. For example, given that the anuran auditory system functions in sexual communication which often occurs in large aggregations, the short, sound-by-sound modulation would be predicted to function in the “on-demand” sorting of ongoing sounds in a chorus. The longer duration modulation, however, could potentially play a role in setting the overall responsiveness of the system relative to: the season; diel cycle; perception of predation risk; an animal's reproductive state and their experience with conspecific and heterospecific signals (Rand et al., 1997; Bee, 2003; Lynch et al., 2006; Miranda and Wilczynski, 2009a,b). The induction of long-term modulation in frogs is comparable to that in other systems (e.g., mammals), as electrical stimuli that are much longer (i.e., minutes to hours) than acoustic stimuli are known to generate auditory modulation that can be measured for several hours (Ma and Suga, 2003, 2005; Zhang and Suga, 2005; Xu et al., 2007; Suga, 2008, 2012; Tang and Suga, 2008; Bajo and King, 2012). Note, however, that in experiments with mammals the duration of modulation is often very long and thus, the categories used to describe its time course (e.g., short and long-term) are based on whether changes can be measured longer or shorter than 3–4 h after electrical stimulation (Suga, 2008). These categories differ from those here, as we operationally defined short-term modulation to occur on a sound-by-sound basis, in which electrical stimulation modulates auditory sensitivity on time scales of less than 1 s (Koch et al., 2011; Mesgarani and Chang, 2012; Lakatos et al., 2013), making the predicted function of “on-demand” modulation possible. Additionally, this faster form of modulation differed from long-term modulation by exhibiting varied modulatory effects, including excitation, inhibition and shifts in latency. Although the mechanisms of these different effects and timing were not assayed, our results suggest a complex of modulatory inputs to the frog ascending auditory system, enabling descending input to either increase or decrease sensitivity.

The second important characteristic of modulation revealed in our study is evidence for stimulus specificity and not simply a generalized modulation of excitability in auditory units. For example, the data showed that modulation of the relative sensitivity of binaural cells to spatially lateralized sounds was capable of shifting ear sensitivity and binaural discriminability (*d*′) by several standards of deviation, effectively shifting a cell's sensitivity to sounds processed only at a single ear. This ear specific modulation is not consistent with a general change in sensitivity because relative (ispi vs. contralateral ear) modulatory effects within cells differed. More evidence against generalized modulation can be found when comparing the probability of modulation between noise and male call stimuli (Table 1). The null hypothesis for a general modulatory effect is that the within-cell probability for modulation to any two sounds should be the same. The data show, however, that modulation of responses to one sound is a poor predictor for another (at least for the sounds used here). Of course, more systematic experiments are needed to understand what acoustic parameters within the complex sounds are being modulated (e.g., frequency specific modulation), including the extent to which modulation is determined by the temporal relationship of the electrical pulse to the envelope and frequency structure of the sound. For example, the time between the electrical pulse and many acoustic elements of the stimuli used here were different. In particular, the acoustic stimuli differed in duration, meaning sounds comprising the end of the calls occurred at varied times after the single electrical pulse. Lastly, with regard to general modulation, the fact that thalamic stimulation caused different modulation of spontaneous and acoustically driven activity is also not consistent with a generalized modulatory effect. The specificity of modulation, especially for sounds eminating from different locations (i.e., lateralization in the headphones), suggests that processing of sound sources could be selectively biased by the frog. Indeed, specificity coupled with the ability to increase and decrease sensitivity means that mechanisms for selective attention potentially exist. This would allow frogs to selectively adjust the relative amount of sensitivity (i.e., attention) assigned to different sources, augmenting peripheral directional filtering to improve communication in large signaling aggregations.

Where might the modulation recorded here occur? It is not clear from our extracellular dataset what circuitry (direct or indirect) is engaged by the electrical stimulus. The modulatory mechanism could include a change in the electrophysiology of the recorded cells (e.g., post-synaptic and/or antidromic) and/or a change at some point pre-synaptic, as there are several auditory synapses peripheral to the TS (Wilczynski and Endepols, 2007), with each potentially being modulated by descending stimulation. In similar experiments using an *in vitro* preparation and intracellular recordings, a train of electrical stimulation to the frog thalamus elicited post-synaptic excitatory and/or inhibitory responses in ipsilateral TS auditory cells at rest (Endepols and Walkowiak, 2000). When TS cells were responding to ascending stimulation of the auditory system, the train of electrical pulses to the thalamus often resulted in reduced auditory sensitivity for up to 2 min. These results are similar to those here for long-term modulation, suggesting that our *in vivo* preparation may be engaging similar post-synaptic mechanisms.

The modulation data in frogs are similar to those in other taxa, including similarities in short-term vs. long-term modulation (Zhou and Jen, 2000; Jen et al., 2002) and location sensitivity (Jen et al., 1998). From a comparative point of view, this raises the possibility that, along with filters mediated in ascending circuitry, descending circuitry that is capable of specific forms of modulation (e.g., specific to source location), may be fundamental to aspects of auditory processing, including source specificity. Past experimental focus on only ascending processing has been due, in part, to the fact that animals can act as feature detectors when responding to functionally relevant stimuli, such that behavior may be elicited by only a narrow subset of stimuli with little response variance (Tinbergen, 1951; Ewert, 1980; Martin, 1994). Consequently, the underlying neural mechanisms mediating the stimulus responses were predicted to be narrowly tuned and static. This prediction has been useful, frequently guiding research that shows neural sensitivity matched to that for behavior (Knudsen and Konishi, 1978; Capranica and Moffat, 1983; Megela Simmons, 2012). However, static auditory filtering may be an exception, even in frogs. Indeed, anatomical data across vertebrates show extensive descending input on to the ascending auditory system (Feng and Lin, 1991; Suga and Ma, 2003; Wilczynski and Endepols, 2007; Bajo and King, 2012). When taken together with electrophysiological manipulation of descending input, it is clear that there is great potential for significant modulation of the ascending code for various acoustic parameters (Suga et al., 2003; Suga, 2008, 2012). Our data, are consistent with this comparative inference and confirm *in vitro* measures (Endepols and Walkowiak, 2000) suggesting significant capabilities for modulating functional auditory codes in a non-mammalian model.

## Author contributions

Abhilash Ponnath and Hamilton E. Farris designed the experiments; collected and analyzed the data; and wrote the paper.

### Conflict of interest statement

The authors declare that the research was conducted in the absence of any commercial or financial relationships that could be construed as a potential conflict of interest.
