# Supplementary material for: Sound-by-sound thalamic stimulation modulates midbrain auditory excitability and relative binaural sensitivity in frogs
Source: Front Neural Circuits. 2014 Jul 25;8:85. doi: 10.3389/fncir.2014.00085 (PMC4111082; doi:10.3389/fncir.2014.00085)
Supplement: Supplementary file 2 [file Presentation2.PDF]

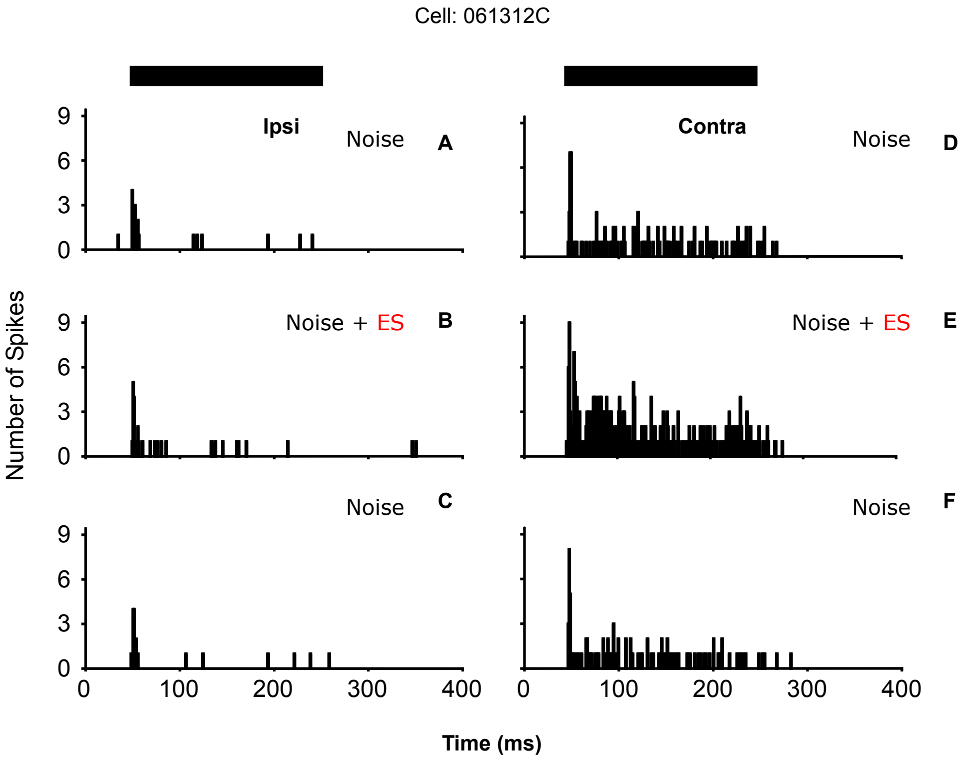

Supplementary Material 2. Figure shows the histograms of the responses of a binaural sensitive TS cell to alternating dichotic acoustic stimulation. Noise bursts (200 ms duration, 20 repetitions to each ear) are presented to the ipsi- (A-C) and contralateral (D-F) ears using headphones. B and E show that the effect of thalamic electrical stimulation is 'ear-specific', as the modulatory effect is different for the responses to the two sound sources. Only the contralateral modulation is significant.
